# Mechanobiological Regulation of Alveolar Bone Remodeling: A Finite Element Study and Molecular Pathway Interpretation

**DOI:** 10.3390/biom16010150

**Published:** 2026-01-14

**Authors:** Anna Ewa Kuc, Magdalena Sulewska, Kamil Sybilski, Jacek Kotuła, Grzegorz Hajduk, Szymon Saternus, Jerzy Małachowski, Julia Bar, Joanna Lis, Beata Kawala, Michał Sarul

**Affiliations:** 1 Department of Dentofacial Orthopedics and Orthodontics, Wroclaw Medical University, 50-425 Wroclaw, Poland; 2 Department of Periodontal and Oral Mucosa Diseases, Medical University of Bialystok, ul. Waszyngtona 13, 15-269 Bialystok, Poland; 3 Faculty of Mechanical Engineering, Military University of Technology, 00-908 Warsaw, Poland; 4 Chair and Department of Oral Surgery, Medical University of Lublin, Witolda Chodźki 6 Street, 20-093 Lublin, Poland; 5 Department of Immunopathology and Molecular Biology, Wroclaw Medical University, 50-556 Wroclaw, Poland; 6 Department of Integrated Dentistry, Wroclaw Medical University, 50-425 Wroclaw, Poland

**Keywords:** tensile microstrain, mechanotransduction, alveolar bone modelig, periodontal ligament biomechanics, molecular pathway, Bone Protection System

## Abstract

**Background:** Mechanical loading is a fundamental regulator of bone remodelling; however, the mechanotransduction mechanisms governing alveolar bone adaptation under tensile-dominant orthodontic loading remain insufficiently defined. In particular, the molecular pathways associated with tension-driven cortical modelling in the periodontal ligament (PDL)–bone complex have not been systematically interpreted in the context of advanced biomechanical simulations. **Methods:** A nonlinear finite element model of the alveolar bone–PDL–tooth complex was developed using patient-specific CBCT data. Three loading configurations were analysed: (i) conventional orthodontic loading, (ii) loading combined with corticotomy alone, and (iii) a translation-dominant configuration generated by the Bone Protection System (BPS). Pressure distribution, displacement vectors, and stress polarity within the PDL and cortical plate were quantified across different bone density conditions. The mechanical outputs were subsequently interpreted in relation to established mechanotransductive molecular pathways involved in osteogenesis and angiogenesis. **Results:** Conventional loading generated compression-dominant stress fields within the marginal PDL, frequently exceeding physiological thresholds and producing moment-driven root displacement. Corticotomy alone reduced local stiffness but did not substantially alter stress polarity. The BPS configuration redirected loads toward a tensile-favourable mechanical environment characterised by reduced peak compressive pressures and parallel (translation-dominant) displacement vectors. The predicted tensile stress distribution is compatible with activation profiles of key mechanosensitive pathways, including integrin–FAK signalling, Wnt/β-catenin–mediated osteogenic differentiation and HIF-1α/VEGF-driven angiogenic coupling, suggesting a microenvironment that may be more conducive to cortical apposition than to resorption. **Conclusions:** This study presents a computational–molecular framework linking finite element–derived tensile stress patterns with osteogenic and angiogenic signalling pathways relevant to alveolar bone remodelling. The findings suggestthat controlled redirection of orthodontic loading toward tensile domains may shift the mechanical environment of the PDL–bone complex toward conditions associated with osteogenic than resorptive responses providing a mechanistic basis for tension-induced cortical modelling. This mechanobiological paradigm advances the understanding of load-guided alveolar bone adaptation at both the tissue and molecular levels.

## 1. Introduction

### 1.1. Biomechanical and Biological Background

Mechanical loading plays a central role in regulating the behaviour of the alveolar bone–PDL complex, where the balance between tensile and compressive stress determines whether tissues undergo modelling or maladaptive remodelling [1,2,3]. When external forces generate excessive localised compression, the PDL may experience hyalinization, microvascular compromise, and activation of osteoclastic pathways, ultimately contributing to root surface resorption and weakening of the adjacent cortical structures [4,5,6]. These biological responses highlight the importance of identifying loading conditions that maintain strains within a physiologic, adaptation-supporting window. Under many commonly used loading configurations, tooth displacement tends to follow a tipping-dominant pattern, producing steep stress gradients concentrated near the cervical and mid-root regions [7,8,9]. Such heterogeneous stress fields create a mechanically unfavourable environment in which compressive peaks exceed adaptive thresholds. In contrast, tensile stress has been associated with upregulation of matrix synthesis and pro-osteogenic signalling, suggesting that stress polarity is a critical determinant of alveolar bone response [10,11]. Achieving loading patterns that reduce compressive biases while enhancing tensile components may therefore be key to promoting modelling rather than resorption.

Large-magnitude displacement requirements can intensify these nonuniform stress distributions, amplifying regions exposed to compressive overload and increasing the probability of mechanically driven bone loss [12,13]. Because the alveolar bone–PDL complex is mechanically heterogeneous—with varying stiffness, geometry, and fibre orientation—small changes in load direction or root trajectory can dramatically alter microstrain patterns. As a result, cortical stress polarity (tension vs. compression) becomes tightly coupled to cellular mechanotransduction, with distinct pathways activated under each condition [14,15]. Numerous biologically oriented approaches have been proposed to modulate tissue turnover; however, most do not directly modify the mechanical input responsible for generating stress distributions across the PDL-bone system. Biological acceleration techniques may increase remodelling capacity, but the final outcome still depends on the spatial distribution of mechanical cues driving osteoclastic or osteoblastic activity. Thus, a mechanobiological framework capable of redirecting load toward tensile-dominant regions is required to clarify how specific stress environments may shift the tissue response from resorptive to osteogenic.

### 1.2. Concept and Design of the Bone Protection System (BPS)

The Bone Protection System (BPS) was developed to address these mechanobiological requirements by integrating controlled force-vector redirection, targeted cortical bone preparation, with the aim of engaging molecular pathways associated with regeneration and tissue synthesis. Rather than relying on artificial augmentation, BPS is intended to harness endogenous signalling cascades that are thought to be triggered by tensile stress within the periodontal ligament (PDL) and trabecular bone, thereby creating conditions that may favour osteogenic activity and matrix deposition. Unlike previous acceleration strategies, which primarily aimed to increase the rate of structural turnover, BPS has been conceived as a mechanobiological paradigm in which precisely shaped loading conditions are combined with local tissue activation to enable stress-guided bone modelling and regeneration.

The objective of this study is to evaluate the mechanical and biological advantages of the BPS loading concept compared to conventional loading configurations, both with and without cortical bone microperforation, using high-resolution finite element method (FEM) modelling. Additionally, its translational potential in regenerative engineering was analysed by investigating conditions under which tensile-stress–dominant loading alone may stimulate bone formation in the absence of additional external forces. A key focus of this work is the exploration of stress-dependent ranges that may favour angiogenesis and osteogenesis within the PDL, by correlating pressure and stress profiles with mechanotransductive pathways reported in the literature. By offering a mechanically guided and biologically driven framework for cortical plate preservation and regeneration, the BPS concept bridges load-based mechanobiology with tissue engineering principles.

## 2. Materials and Methods

In present study, it was very important to maintain exactly the same conditions for each of the variants analysed during the research. In addition, it was very important to be able to perform measurements that cannot be conducted on a real patient. Therefore, the decision was made to utilise a research method based on the Finite Element Method (FEM)

However, FEM-based studies require the development of a very accurate numerical model that will faithfully reflect the actual conditions. For the purposes of the study described in the article, an advanced FEM model was developed using real patient data.

**Figure 1:**
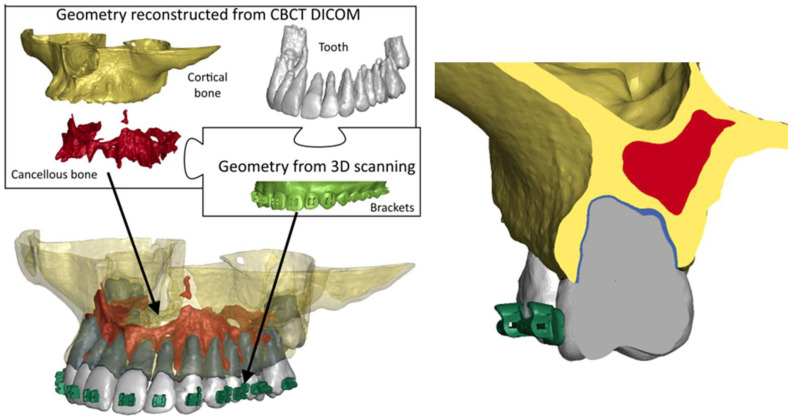
FE model and cross-section of the model.

First, cortical bone, cancellous bone, and teeth were manually outlined on DICOM images containing CBCT imaging. Then, 3D models were reconstructed from cross-sections on individual layers (Figure 1). This was done using MIMICS 18.0 software (Materialise, Leuven, Belgium) [16]. Next, the geometry of the brackets was recorded using an intraoral 3D scanner. Using the geometry of the teeth and bone, the geometry of the PDL was determined using Boolean operations. Based on the literature [17]. It was assumed that the PDL has a constant thickness of 0.25 mm. The geometries obtained in this way were used to prepare a finite element mesh in the Hypermesh system (Altair, Troy, MI, USA). The geometry of the bone is very complex, so it was decided to use tetragonal elements with an average side length of 0.3 mm to represent it. The same approach was used for teeth and locks. PDL, on the other hand, was modelled using hexagonal elements with a side length of 0.25 mm. The final model is shown in Figure 1.

In the numerical model, it was confirmed that the brackets, teeth and bones would be subjected to minor loads, with no significant stress occurring in these elements [18]. Consequently, these structures were modelled using an isotropic, linear elastic constitutive model. In turn, bone stiffness is a fairly important parameter in the model. Previous work by the authors [19] shows that compact bone stiffness significantly affects the loads in the PDL and the behaviour of the entire model. Hence Young’s modulus of cortical bone was varied between 12.5 GPa and 27.5 GPa in increments of 3.0 GPa, with extreme values derived from the literature [16]. The brackets were made of steel. The material data for the above components are given in Table 1.

The component subjected to the highest load—and also the primary focus of the authors—was the periodontal ligament (PDL). Therefore, the hyperelastic Ogden model was used to model the PDL. This model assumes that the behaviour of the material can be described by the strain energy function, from which the stress–strain relationship can be derived. In the Ogden model, the strain energy function is defined by the function:

(1) $$W=\sum_{i=1}^{3}\sum_{j=1}^{n}\frac{\mu_{j}}{\alpha_{j}}\left(\lambda_{i}^{\alpha }j-1\right)+K\left(J-1-\ln J\right),$$

where W is the strain energy potential, λi is the main deviant stretches, µi and αi are material parameters, J is the determinant of the elastic strain gradient, and K is the volume modulus. The bulk modulus is calculated using the values of Poisson’s ratio and Young’s Modulus. The parameters presented in Table 2 [17] were used to describe the behaviour of the PDL.

To reproduce connections between individual components a TIED-type contact was used between the structures that are connected (cancellous bone, cortical bone, PDL, tooth, and brackets).

During the calculations of the upper surface of the skull base, all degrees of freedom were constrained. Previous studies by the authors have shown that such a distance between the anchorage and the PDL has a positive effect on the reproduction of the actual behaviour of the model within the teeth and surrounding structures. Numerical calculations were performed in the LS-Dyna system using an implicit integration step. Three main scenarios were analysed during the numerical simulations:

C1: Conventional expansive orthodontic force (100 g per tooth) without corticotomy.

C2: Orthodontic force (100 g per tooth) with corticotomy alone (3 mm deep cuts in each interradicular space and 3 mm above the root apices)

C3: Full Bone Protection System (BPS) protocol (orthodontic force 100 g per tooth combined with 3 mm corticotomy and temporary anchorage devices [TADs] applying a buccal force vector).

The orthodontic force magnitude was set to 100 g per tooth, which lies within the range commonly used clinically for controlled anterior translation and intrusion in adult patients. This value was chosen to provide a realistic loading condition while allowing direct comparison between the three configurations under identical external load. For the assumed load level, the PDL, teeth and bone structures work within a linear range, and the stress value is directly proportional to the applied force. The goal of the present analysis was therefore not to determine an optimal force magnitude, but to examine how different loading (force, BPS) and structure (corticotomy cuts) configurations redistribute stress and displacements within the PDL–bone complex for a choosen, clinically relevant load.

**Figure 2:**
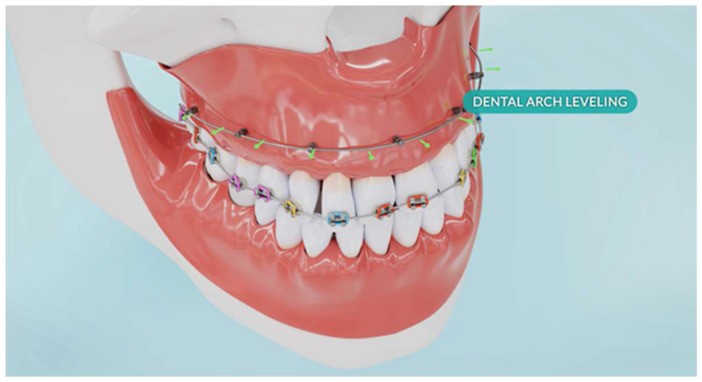
Geometric configuration of the Bone Protection System used for finite element modelling.

For the BPS protocol, corticotomy was simulated with incisions made in every other interradicular space, using piezoelectric cuts extending to the cancellous bone. In the numerical model, this was represented by removing finite elements in the volume covering the incisions. In interradicular spaces without incisions, orthodontic mini-implants (TADs) were placed reflected in the model through the use of CNRB elements), and an expansive force was applied (Figure 2) (by defining a force vectors perpendicular to the surfaces of contact between the brackets and the tooth).

Finaly, 18 numerical models were developed (6 variants of compact bone stiffness × 3 load variants).

## 3. Results

### 3.1. Pressure and Stress Distributions

**Figure 3:**
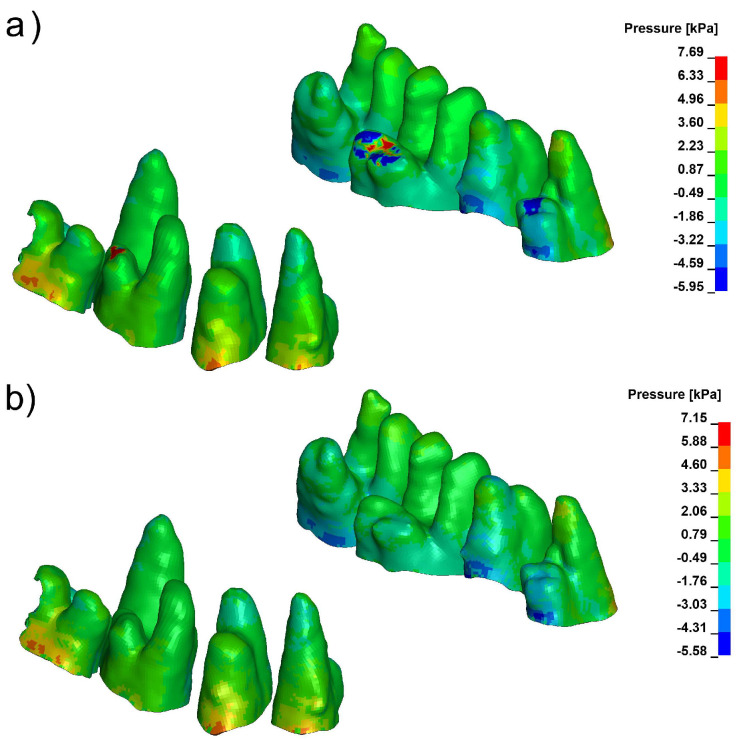
Pressure in marginal periodontium without corticotomy E = 12,500 (**a**), E = 27,500 (**b**) (positive values indicate compression, negative values indicate tension).

Pressure and stress analyses revealed distinct differences in the spatial distribution of periodontal loading across the three evaluated configurations. Under conventional loading, the finite element model consistently produced a compression-dominant environment in the marginal periodontal ligament (PDL), with peak hydrostatic pressures frequently exceeding 9 kPa in high-density bone (E = 27,500 MPa). These elevated compressive values were concentrated mainly in the cervical and mid-root regions, particularly on the vestibular side, as illustrated in Figure 3.

**Figure 4:**
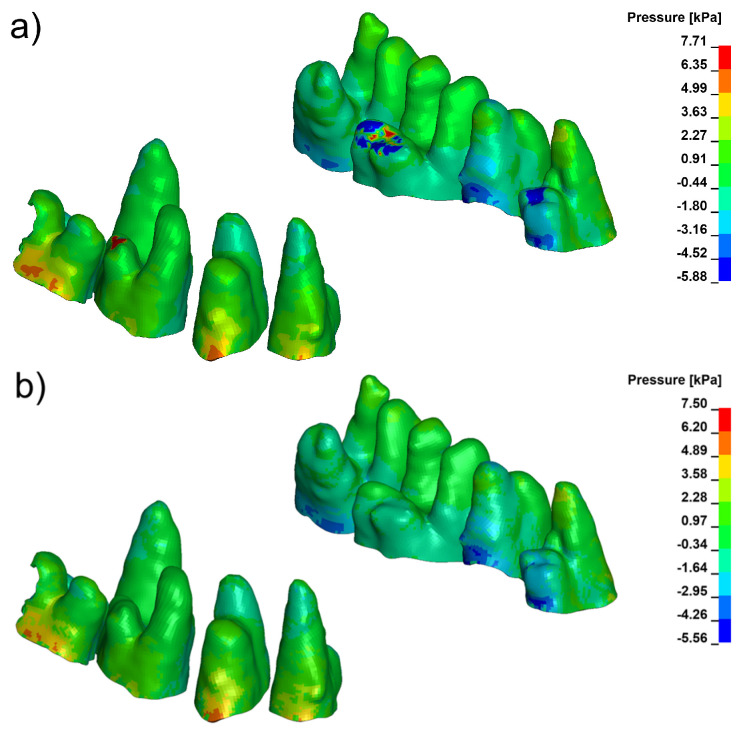
Pressure in marginal peridontium with 3 mm depth corticotomy (**a**) E = 12,500, (**b**) E = 27,500 (positive values indicate compression, negative values indicate tension).

Corticotomy alone did not substantially modify the magnitude or distribution of this compressive stress. Although the 3 mm cortical microperforation reduced local stiffness of the vestibular plate, the mechanical environment within the PDL remained compression-biassed, and the overall pattern of pressure distribution was similar to that seen under baseline loading (Figure 4).

**Figure 5:**
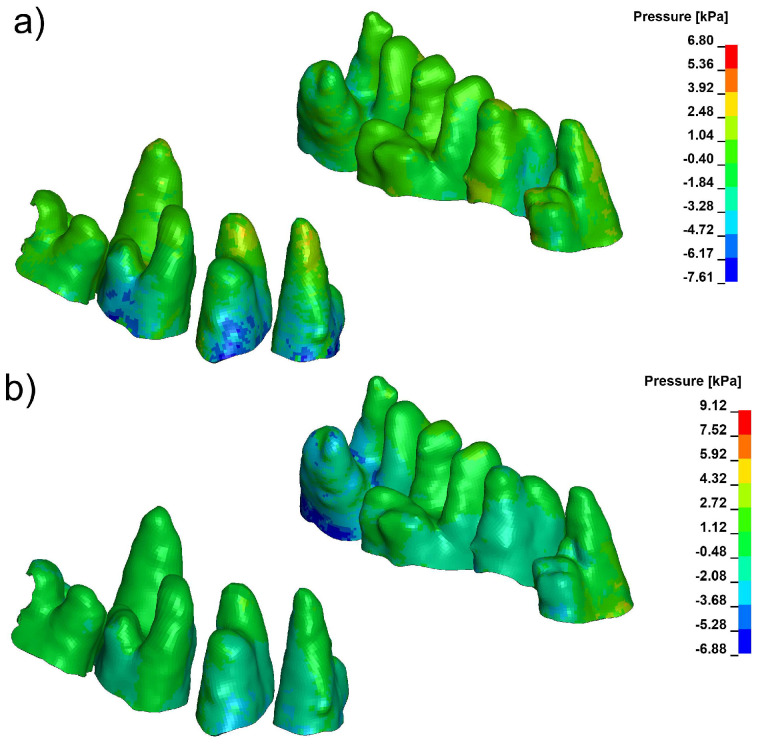
Pressure in marginal periodontium with Bone Protection System (**a**) E = 12,500, (**b**) E = 27,500 (positive values indicate compression, negative values indicate tension).

In contrast, the Bone Protection System (BPS) produced a marked redistribution of stress throughout the PDL–bone complex. Across both cortical density settings, BPS decreased peak compressive pressures in the marginal PDL and shifted the overall pressure distribution toward tensile-favourable loading. Distinct tensile-favourable pressure regions were consistently observed along the vestibular aspect of the root and cortical plate (Figure 5). Quantitative values for peak hydrostatic pressure in the marginal PDL for all configurations and density settings are summarised in Table 3 and Table 4.

### 3.2. Displacement Patterns and Load Directionality

**Figure 6:**
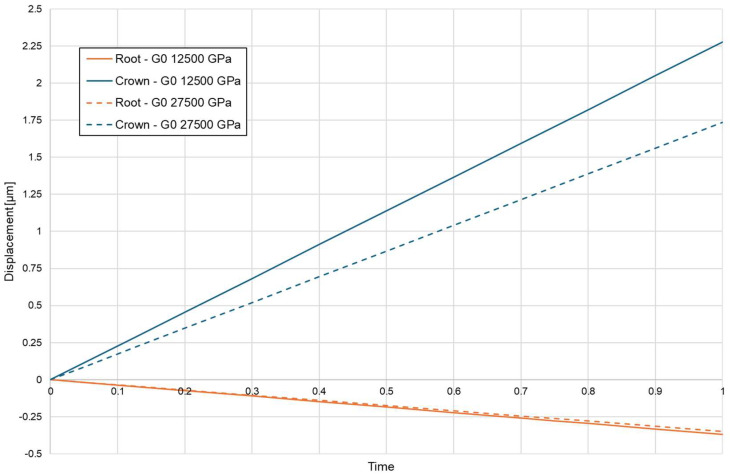
Coronal—blue and apical—orange displacement without corticotomy E = 12,750, E = 27,500.

Directionality of root displacement also differed substantially among the loading conditions. Conventional loading produced a moment-driven trajectory, characterised by a clear divergence between apical and coronal displacement vectors along the root. This bending-dominant response generated a steep stress gradient across the PDL. The magnitude of this divergence was strongly influenced by cortical bone density, with higher-density bone producing larger differences between apical and coronal displacement components (Figure 6).

**Figure 7:**
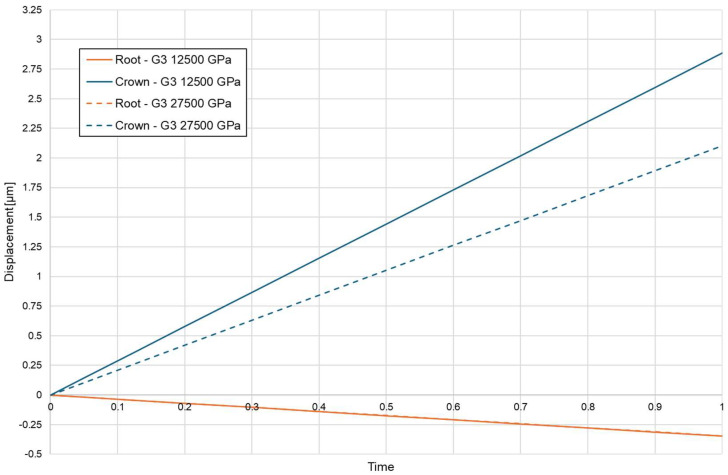
Coronal—blue and apical—orange displacement with 3 mmm depth corticotomy E = 12,750, E = 27,500.

Cortical microperforation altered displacement amplitude but did not fundamentally change the polarity of loading. Under corticotomy-assisted conditions, overall displacement increased; however, displacement vectors remained non-parallel, and the PDL continued to experience a bending-dominated pattern with persistent cervical compression (Figure 7).

**Figure 8:**
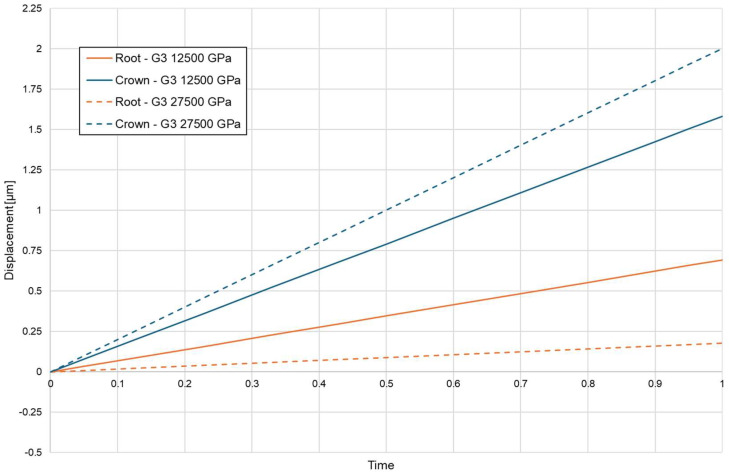
Coronal—green and apical—red displacement with Bone Protection System E = 12,750, E = 27,500.

In contrast, the BPS configuration substantially altered the load-transfer pathway. The translation-oriented mechanics of BPS redirected forces in a way that reduced bending moments and generated a predominantly axial displacement pattern, with parallel vector orientation across the root surface (Figure 8). The extent of apical displacement depended on bone density and was greater in low-density bone, but the directional coherence of the displacement field was preserved across both density settings. Table 5 summarises apical and coronal displacement magnitudes for all configurations.

### 3.3. Summary of Mechanical Differences Between Configurations

Taken together, the simulations indicate that:

- Conventional loading concentrates high compressive stress in the marginal PDL and produces moment-driven displacement with pronounced apico-coronal divergence;
- Corticotomy alone increases displacement amplitude but does not substantially reduce marginal compression or convert the bending-dominant response into translation;
- BPS reduces peak compressive stress in the marginal PDL, introduces well-defined tensile-favourable regions along the vestibular PDL and cortical plate, and generates a translation-dominant displacement pattern with aligned crown-root vectors.

These findings show that modifying load direction through skeletal anchorage and controlled force redirection can transform a compression-dominated mechanical environment into one with reduced compressive overload, more homogeneous displacement and a tensile-favourable stress distribution in regions prone to cortical thinning and modelling.

## 4. Discussion

### 4.1. Mechanical Interpretation

The present patient-specific finite element analysis compared three clinically relevant loading configurations—conventional orthodontic mechanics, corticotomy-assisted loading and a translation-dominant Bone Protection System (BPS)—in terms of their effects on stress distribution and displacement patterns within the PDL–bone complex. The simulations showed that conventional loading consistently produced a compression-dominant marginal environment and a moment-driven displacement pattern, whereas BPS generated a tensile-favourable stress field and translation-oriented root movement. Corticotomy alone increased displacement amplitude but did not fundamentally change the polarity of loading.

Under conventional loading, peak compressive stress in the marginal PDL routinely exceeded 9 kPa in high-density cortical bone, with pressure hotspots localised to cervical and mid-root regions. This pattern was accompanied by a bending-dominated displacement trajectory with divergent apical and coronal vectors. Such a configuration is mechanically efficient for producing tipping and rotation, but it also concentrates mechanical load in thin vestibular cortices and may predispose to excessive local compression in anatomically vulnerable areas.

Corticotomy was expected to reduce cortical stiffness and thereby modify the load-bearing behaviour of the alveolar plate. In the present model, however, a 3 mm vestibular corticotomy did not substantially alter peak compressive stress or the overall pattern of PDL loading. Displacement amplitude increased, but displacement vectors remained non-parallel, indicating that the system still behaved predominantly as a bending lever. These findings suggest that corticotomy, when used without deliberate modification of the external force system, may not be sufficient to convert a compression-dominant regime into a more favourable translation-dominated one. In other words, reducing cortical stiffness alone does not guarantee a protective loading environment for the marginal PDL.

In contrast, the BPS configuration combined skeletal anchorage with controlled force redirection to fundamentally change the load-transfer pathway. The simulations showed that BPS reduced bending moments, aligned apical and coronal displacement vectors, and introduced well-defined tensile-favourable pressure regions along the vestibular PDL and cortical surface. Importantly, this occurred across all simulated density conditions, although the absolute magnitude of displacement depended on bone stiffness.

From a biomechanical perspective, BPS can therefore be viewed primarily as a load-redistribution strategy rather than a simple increase or decrease in force magnitude. By shifting the vector of action towards controlled translation and by engaging skeletal anchorage, BPS unloads the most vulnerable marginal cortical areas and re-orients stress into a regime in which tensile components play a greater role. This suggests a potential mechanism by which BPS might reduce the risk of marginal overload and root concentration of compressive stress in thin or high-density cortical regions, particularly in patients with a fragile periodontal phenotype.

Although this study is numerical, the mechanical patterns observed have clear implications for clinical biomechanics. Moment-driven loading with concentrated marginal compression is more likely to challenge the limits of the alveolar housing in patients with thin buccal cortices and diminished bone volume. In such cases, strategies that promote translation-dominant movement and distribute stress more evenly—such as BPS-type skeletal anchorage—may provide a safer mechanical envelope for tooth movement, even at similar nominal force levels.

At the same time, the present model is based on a single patient-specific anatomy and evaluates a single nominal force magnitude (100 g per tooth). The results should therefore be interpreted as illustrating mechanistic tendencies rather than defining universal thresholds or population-level averages. Interindividual differences in root morphology, cortical thickness, bone density and periodontal phenotype are likely to modulate both the absolute values and spatial patterns of stress. In particular, thin buccal plates and thin periodontal phenotypes, with reduced cortical thickness and soft-tissue volume, provide less structural buffering and can be expected to exhibit higher and more localised marginal compressive peaks under conventional loading, whereas thicker cortices and more robust root forms may distribute the same load over a larger area with lower local stress concentrations. Further modelling work incorporating parametric variation and cohort-based geometries will be necessary to test how robust the observed BPS-related advantages are across a wider clinical population.

**Figure 9:**
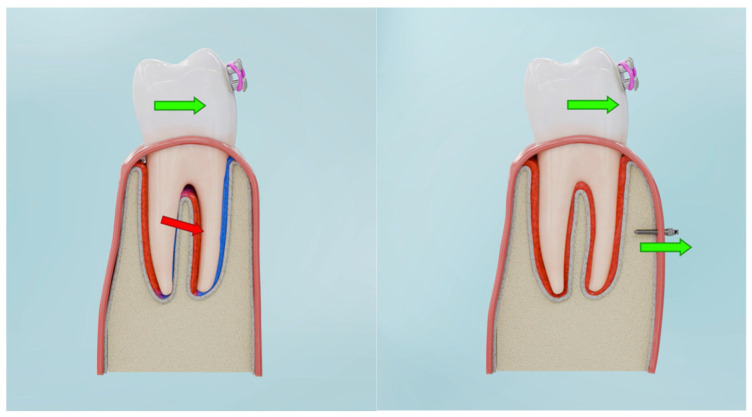
Conversion of compressive zones (blue) into tensile zones (red) under BPS-guided loading.

From a clinical perspective, BPS differs from existing concepts such as corticotomy-assisted orthodontics and Periodontally Accelerated Osteogenic Orthodontics (PAOO), which primarily aim to increase remodelling capacity without fundamentally altering the direction of the force system. Similarly, conventional TAD-based mechanics are often used to reinforce anchorage or allow en-masse retraction, but they do not necessarily generate a translation-dominant, tensile-favourable environment in the marginal PDL. In contrast, BPS was specifically designed as a load-redistribution strategy, in which skeletal anchorage and cortical preparation are combined to shift stress polarity from cervical compression towards vestibular tensile strain. The transition from compression-dominant to tension-favourable zones under BPS is illustrated conceptually in Figure 9.

### 4.2. Mechanobiological Hypothetical Interpretation of Bone Regeneration Under BPS

Importantly, the mechanotransductive pathways discussed in this section are drawn from established experimental and clinical literature on periodontal and bone biology, rather than inferred de novo from the present FEM simulations and should be viewed as hyphothesis-generating].

The Bone Protection System (BPS) was designed to address a key limitation of conventional loading mechanics: the lack of biological protection for vulnerable cortical bone during mechanically induced displacement. By redirecting forces to generate controlled buccal distraction, BPS applies tensile stress to the vestibular cortical plate while maintaining patency of both palatal and buccal periodontal ligaments for vascular flow and molecular signalling. Experimental work has shown that such deformation and fluid shear can activate mechanosensitive pathways, including integrin-FAK, Wnt/β-catenin and MAPK signalling, which support osteogenic differentiation and matrix synthesis [20,21,22,23]. In this context, the tensile-favourable microstrain environment predicted by our FEM model under BPS provides a plausible mechanical substrate for tension-driven bone adaptation.

**Figure 10:**
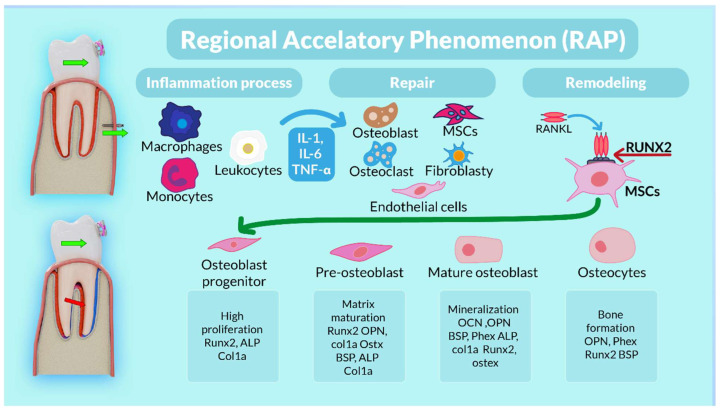
Schematic representation of load-driven tooth displacement and subsequent bone repair via regenerative processes involving different cell types, such as immune cells, endothelial cells and mesenchymal stem cells (MSCs) capable of differentiating into osteoblasts. Steps in the osteogenic differentiation of MSCs: MSCs actively proliferate during the initial stages of osteogenesis and produce collagen; they then start expressing osteogenic markers such as alkaline phosphatase (ALP), secreted by early osteoblasts (matrix maturation phase). In the final stage, high expression of osteocalcin and osteopontin, secreted by late osteoblasts, is observed, followed by calcium and phosphate deposition (mineralisation phase). In the bone-forming phase, osteoblasts become osteocytes. Runx2, runt-related transcription factor 2; ALP, alkaline phosphatase; Col1a1, collagen type I alpha 1; Osterix, Ostx; OPN, osteopontin; BSP, bone sialoprotein; Phex, phosphate-regulating endopeptidase homologue; OCN, osteocalcin [40,41,42].

Corticotomy (Figure 10) initiates a Regional Acceleratory Phenomenon (RAP) characterised by transient upregulation of RANKL, IL-1β and TNF-α, promoting osteoclast activation, together with increased VEGF and HIF-1α expression, which enhance angiogenesis [20,21,22,23,24,25,26,27]. Concurrently, BMP-2, TGF-β and IGF-1 signalling stimulates osteoblast recruitment and activity, loosening the cortical matrix and increasing its susceptibility to remodelling [20]. Within this activated environment, BPS mini-implants provide a stable anchorage platform for controlled buccal distraction of the cortical plate (Figure 1 and Figure 2). The resulting mechanical microtrauma further upregulates HIF-1α and VEGF, increases vascular permeability and facilitates recruitment of progenitor cells such as CD34 + cells and osteoprogenitors [21]. Together, these events favour a shift in the RANKL/OPG balance and bone turnover dynamics toward bone formation rather than resorption [21,22].

Several aspects of BPS-mediated loading resemble mandibular distraction osteogenesis (MDO), in which bone and surrounding soft tissues are stretched by a controlled mechanical vector. Classical distraction osteogenesis comprises latency, distraction and consolidation phases, each associated with distinct molecular profiles [24]. In the latency phase, activation of the TGF-β pathway promotes mesenchymal stem cell (MSC) proliferation and induces expression of Runx2, initiating osteoblast differentiation [24]. Increased COL1A1 expression signals the onset of extracellular matrix synthesis, whereas during the distraction phase, bone stretching activates MAPK/ERK signalling and Wnt/β-catenin pathways, supporting osteoblast proliferation, survival and osteogenic gene expression [25,26]. Local hypoxia further stabilises HIF-1α and upregulates VEGF, coupling angiogenesis to new bone formation [27]. In the consolidation phase, mineralisation and maturation predominate, with increased osteocalcin (OCN) and osteopontin (OPN) expression and sustained BMP-2–Smad signalling [27,28]. Immune cells, including macrophages and T lymphocytes, also contribute by modulating inflammation and secreting cytokines and interleukins that influence bone remodelling, underscoring the importance of an immunological–osseous axis in distraction-induced regeneration [29].

Orthodontic tooth movement is itself a form of mechanically induced bone repair. Mechanical loading of the PDL–bone complex triggers a sequence of lag, movement and remodelling phases that involve tightly coupled osteoclastic resorption and osteoblastic formation [30,31,32,33,34,35]. Bone repair can be viewed as a regenerative response to microdamage, in which newly formed bone gradually restores structural and physiological continuity with surrounding tissue [33,35,36,37]. Experimental studies have shown that this process depends on coordinated activity of osteoblasts, osteoclasts, osteocytes, MSCs and endothelial cells, which together maintain the balance between bone resorption and formation and preserve bone homeostasis [33,37]. Early phases of tooth movement are characterised by an acute inflammatory response with upregulation of cytokines such as IL-1, IL-6, IL-11, IL-18 and TNF-α, which recruit macrophages and neutrophils to the injured region [38]. These cells secrete fibroblast growth factor-2 (FGF2) and insulin-like growth factors (IGFs), contributing to early haematoma formation and granulation tissue [39]. Additional mediators, including TGF-β and platelet-derived growth factor (PDGF), stimulate osteoprogenitor cells to release bone morphogenetic proteins (BMPs) and further drive osteogenic differentiation [28]. Both innate and adaptive immune responses, together with MSC-derived immunomodulatory signals, influence osteoclastic activity, bone resorption rate and the pace of tooth movement [30,31,35]. Increasing evidence suggests that osteoblast-mediated bone apposition plays a central role in determining the long-term pattern of orthodontic bone adaptation [31].

Within this broader mechanobiological framework, BPS can be interpreted as a load-redistribution strategy that combines RAP-induced tissue activation with a tensile-biassed stress field. By unloading the marginal cortical plate through buccal expansion and translation-dominant root displacement—rather than tipping—BPS reduces excessive cervical compression, preserves cortical integrity and creates a tensile-favourable environment consistent with strain-guided bone modelling and distraction-like regeneration [21,22,23,33,35]. While the present FEM study does not directly measure these molecular events, the simulated stress and displacement patterns are compatible with known pathways of osteogenesis and angiogenesis under tension-driven loading [20,21,22,23,24,25,26,27,28,29,33,34,35,36,37,38,39,40,41,42,43,44]. (Figure 10).

The analysis indicates that the efficacy of the Bone Protection System (BPS) is contingent on both biomechanical and biological factors, including the magnitude of force, miniimplant depth, bone density, archwire activation, and the technique and depth of corticotomy incisions. Translational force vectors that induce tensile rather than compressive stress correlate with optimal bone remodelling. The root length and crown-to-root ratio significantly influence BPS efficacy. Biomechanically, longer roots facilitate axial movement with BPS, whereas shorter roots are more prone to uncontrolled tipping.

Also interindividual differences in root morphology, cortical thickness, bone quality and periodontal phenotype may substantially influence local stress distribution.

Although no new clinical or histological data are presented in this manuscript, the BPS loading concept analysed here has already been applied in a small number of clinical proof-of-concept cases with CBCT-documented preservation or apposition of the buccal cortical plate around mandibular and maxillary incisors. These cases, reported separately in dedicated clinical phenotype articles, showed soft-tissue thickening and absence of cortical breakdown under BPS-modulated mechanics. While these observations are preliminary and limited in number, they qualitatively agree with the present FEM-predicted shift from cervical compression toward a tensile-favourable environment in the marginal PDL.

The current study should therefore be viewed as providing the mechanical and mechanobiological ‘backbone’ of an emerging clinical concept, complementing rather than replacing the clinical validation and CBCT-based observations reported in companion manuscripts. The bigger research is in progress with the bioethical agreement.

## 5. Limitations

This study has several limitations that should be considered when interpreting the findings. First, the finite element model is based on a single patient-specific CBCT dataset. Although this increases anatomical realism, it limits generalizability, as variation in root morphology, cortical thickness, bone quality and periodontal phenotype may substantially influence local stress distributions. Future work should therefore incorporate parametric modelling and cohorts representing different morphologies and phenotypes.

Second, only one nominal orthodontic force level (100 g per tooth) was simulated. Within the elastic range of materials, stress and displacements scale approximately proportionally with load magnitude; however, a full force-sensitivity analysis was beyond the scope of this work. Our conclusions should therefore be viewed as comparing relative differences between loading configurations under a fixed, clinically plausible load, rather than defining an optimal force level.

Third, the mechanobiological interpretation is based on superimposing FEM-derived stress patterns onto molecular and cellular responses reported in independent in vitro and in vivo studies. We did not obtain direct molecular, histological or perfusion data in the present work. As a result, the proposed mapping between tensile- and compression-dominant zones and specific signalling pathways should be regarded as hypothesis-generating rather than confirmatory.

Fourth, although the main analysis focuses on a single representative BPS loading configuration, this configuration was not selected arbitrarily. During the design phase, we conducted preliminary exploratory simulations using simplified single-root models, in which mini-implant insertion depth and vertical position relative to the alveolar crest were systematically varied. The purpose of these exploratory tests was to identify clinically feasible anchorage geometries capable of generating coherent, translation-dominant displacement patterns and tensile-favourable stress fields while avoiding excessive local stress concentrations. These simulations served as a qualitative screening step rather than a comprehensive parametric sensitivity analysis, and their detailed results are therefore not reported here. Consequently, the present study does not aim to define the full mechanical design space of BPS-like systems, and future FEM investigations should formally quantify how variations in implant depth, vertical positioning, and inter-implant spacing influence stress distribution and displacement coherence.

Finally, some of the authors are listed as inventors on a pending patent related to the Bone Protection System. This patent filing serves to protect the intellectual contribution underlying the proposed mechanical concept and to prevent uncontrolled or premature clinical implementation. The primary motivation of this work is scientific and clinical: to reduce the risk of periodontal and alveolar complications during orthodontic treatment by improving the biomechanical environment of the supporting tissues. Independent replication, external validation, and prospective clinical trials will be essential to substantiate the mechanobiological implications suggested by this model.

## 6. Conclusions

This study presents a computational–molecular framework linking finite element–derived tensile stress patterns with osteogenic and angiogenic signalling pathways relevant to alveolar bone remodelling. The findings suggestthat controlled redirection of orthodontic loading toward tensile domains may shift the mechanical environment of the PDL–bone complex toward conditions associated with osteogenic than resorptive responses providing a mechanistic basis for tension-induced cortical modelling. This mechanobiological paradigm advances the understanding of load-guided alveolar bone adaptation at both the tissue and molecular levels.

## Figures and Tables

**Table 1:** Material data used to describe the material behaviour.

Component	Young’s Modulus [MPa]	Poisson’s Ratio [ ]
Steel	210,000	0.30
Tooth	18,600	0.31
Cortical bone	12,500–27,500	0.30
Cancellous	2000	0.30

**Table 2:** Parameters used for describing the PDL.

*µ*_1_ [MPa]	*α*_1_ [MPa]	Poisson’s Ratio [ ]
2.5 × 10^−3^	150	0.46

**Table 3:** Maximum hydrostatic value in the PDL [kPa].

Load Variant	Young’s Modulus of Cortical Bone [GPa]					
	12.5	15.5	18.5	21.5	24.5	27.5
C1	7.69	7.53	7.40	7.30	7.22	7.15
C2	7.71	7.66	7.62	7.58	7.54	7.50
C3	6.80	7.05	7.75	8.30	8.75	9.12

**Table 4:** Minimum hydrostatic value in the PDL [kPa].

Load Variant	Young’s Modulus of Cortical Bone [GPa]					
	12.5	15.5	18.5	21.5	24.5	27.5
C1	−5.95	−5.84	−5.76	−5.69	−5.63	−5.58
C2	−5.88	−5.79	−5.72	−5.66	−5.61	−5.56
C3	−7.61	−6.43	−5.43	−5.76	−6.36	−6.88

**Table 5:** Apical and coronal displacement [µm].

	Load Variant	Young’s Modulus of Cortical Bone [GPa]					
		12.5	15.5	18.5	21.5	24.5	27.5
Crown displacement	C1	2.28	2.09	1.97	1.87	1.80	1.74
	C2	2.88	2.62	2.44	2.30	2.19	2.10
	C3	1.58	1.73	1.83	1.90	1.96	2.00
Root displacement	C1	−0.37	−0.36	−0.36	−0.36	−0.35	−0.35
	C2	−0.35	−0.35	−0.35	−0.35	−0.35	−0.35
	C3	0.69	0.53	0.41	0.32	0.24	0.18
Difference	C1	2.65	2.45	2.33	2.23	2.15	2.09
	C2	3.23	2.97	2.79	2.65	2.54	2.45
	C3	0.89	1.2	1.42	1.58	1.72	1.82

## Data Availability

The original contributions presented in this study are included in the article. Further inquiries can be directed to the corresponding author.
